# Does a Strong El Niño Imply a Higher Predictability of Extreme Drought?

**DOI:** 10.1038/srep40741

**Published:** 2017-01-17

**Authors:** Shanshan Wang, Xing Yuan, Yaohui Li

**Affiliations:** 1 CAS Key Laboratory of Regional Climate-Environment for Temperate East Asia (RCE-TEA), Institute of Atmospheric Physics, Chinese Academy of Sciences, Beijing 100029, China; 2 Key Laboratory of Arid Climatic Change and Reducing Disaster of Gansu Province, and Key Open Laboratory of Arid Climate Change and Disaster Reduction of CMA, Institute of Arid Meteorology, CMA, Lanzhou 730020, China

## Abstract

The devastating North China drought in the summer of 2015 was roughly captured by a dynamical seasonal climate forecast model with a good prediction of the 2015/16 big El Niño. This raises a question of whether strong El Niños imply higher predictability of extreme droughts. Here we show that a strong El Niño does not necessarily result in an extreme drought, but it depends on whether the El Niño evolves synergistically with Eurasian spring snow cover reduction to trigger a positive summer Eurasian teleconnection (EU) pattern that favors anomalous northerly and air sinking over North China. The dynamical forecast model that only well represents the El Niño underpredicts the drought severity, while a dynamical-statistical forecasting approach that combines both the low- and high-latitudes precursors is more skillful at long lead. In a warming future, the vanishing cryosphere should be better understood to improve predictability of extreme droughts.

The past monster El Niño of 2015/16 resulted in widespread droughts and floods across the globe^1^,^2^,^3^,^4^. For instance, North China suffered extreme drought in the summer of 2015, where two agricultural provinces (Henan and Liaoning) were hit by the worst drought on record since 1951. Indeed, the 2015 North China drought affected 21 million people in seven provinces, influenced 3.4 million hectares and destroyed 0.4 million hectares of crops, which caused direct economic loss of 11.48 billion RMB (http://www.chinaam.com.cn). Such devastating impact raises the question of whether a strong El Niño imply an extreme summer drought in North China, given that an El Niño can be predicted a few months ahead^5^ that might be critical for drought mitigation practices.

In fact, El Niño can boost the chance of droughts across North China as a first-order external forcing^6^,^7^,^8^, via altering the tropical and subtropical circulation anomalies and carrying the influence far to the mid and high latitudes by teleconnection^9^,^10^. However, not all extreme summer droughts in North China occur with strong El Niño-like sea surface temperature (SST) forcings, including the 2014 extreme summer drought^11^. Moreover, a strong El Niño (e.g., 1982/83 and 1997/98) does not necessarily result in an extreme summer drought in North China, while some persistent large-scale atmospheric circulation anomalies are more relevant^12^,^13^. The drought-prone circulation anomalies include a weaker East Asia summer monsoon (EASM)^14^,^15^, enhanced west Pacific subtropical high (WPSH)^16^ and East Asian jet stream^17^, and a positive summer Eurasia teleconnection (EU) pattern with a high pressure anomaly over Lake Baikal^11^,^18^. These circulation anomalies have been widely diagnosed for the causes of summer droughts in North China from the perspectives of atmosphere-ocean interaction^8^,^19^ and land-atmosphere coupling^20^,^21^,^22^. Nevertheless, the dominant circulation pattern that is responsible for extreme summer droughts in North China, and the predictable causes for such circulation pattern, are still unclear.

Since the ocean-atmosphere coupled general circulation models (CGCMs) that make use of oceanic memory (e.g., El Niño SST anomaly) for seasonal forecasting have now been widely used to provide drought early warning^23^,^24^,^25^, it is essential to understand whether a strong El Niño indicates a higher predictability of extreme summer drought in North China. This will help to interpret the dynamical seasonal forecasting products for a better climate service such as drought mitigation.

In present study, we diagnose the cause and predictability of extreme summer droughts in North China based on multisource observational data, and ensemble hindcasts and real-time forecasts from a dynamical seasonal forecast model—Climate Forecast System version 2 (CFSv2)^26^. Our results suggest that the predictability of extreme summer droughts across North China depends on whether the model captures the positive EU pattern, while it is not solely associated with the predictability of El Niño. In fact, the reduction in high-latitudes snow cover also plays a crucial role in triggering such circulation pattern. Along this line, a dynamical-statistical forecasting approach that merges preceding Eurasian spring snow cover and the CFSv2 predicted El Niño is established, and the approach shows more reliable predictions for extreme droughts in North China at long forecast lead.

## Results

### Causes of the 2015 extreme summer drought

**Figure 1:**
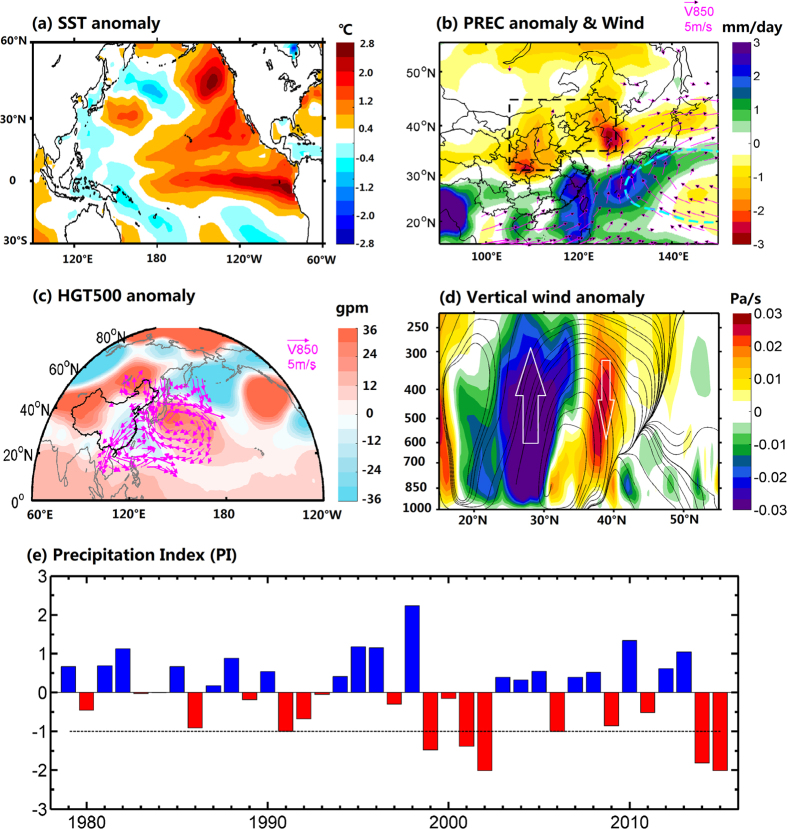
Perspective of the 2015 extreme summer drought. (**a**) SST anomaly (°C) and (**b**) precipitation anomaly (shading, mm/day) with absolute wind currents at 850 hPa averaged during July-August (JA) in 2015. The thick cyan contour is 5880 gpm, implying the location of the west Pacific subtropical high. (**c**) Anomalies of 500 hPa geopotential height (shading, gpm) and 850 hPa wind (vectors, m/s). (**d**) Vertical–meridional cross section averaged along 120°–130°E for the summer vertical wind (streamline) and omega anomalies (shading, Pa/s). (**e**) Time series of Precipitation Index (PI) defined as normalized and area-weighted mean precipitation anomaly averaged over the boxes of (35°N–45°N, 116°E–128°E) and (31°N–45°N, 105°E–116°E) in (**b**). All the anomalies are based on the climatology of 1982–2010. Maps were produced using Matlab version R2012a software (http://www.mathworks.com).

Figure 1a shows that the El Niño was already very powerful during the summer of 2015, and the values of SST anomaly exceeded 1.5–2 °C over a large area of the eastern tropical Pacific Ocean. With the influence of El Niño, North China experienced an extreme summer drought, with precipitation decreased by 30% relatively to the climatology (Fig. 1b). In the summer of 2015, the WPSH was stronger than the climatology but located further east, so a great amount of moisture was transported along the western side of WPSH, from South China Sea, via south China and Taiwan, to southern Japan, resulted in a severe moisture deficit in North China (Fig. 1b). Meanwhile, there were an high pressure anomaly around Lake Baikal and low pressure anomalies in both upstream (Ural Mountains) and downstream (eastern China and its adjacent sea) regions (Fig. 1c), which is similar to the positive summer EU pattern^27^. Such EU pattern resulted in anomalous northerlies between the high- and low-pressure centers over North China, weakened the EASM, and thereby reduced the moisture transported from the south. In addition to the moisture deficit, there was a negative descending motion in North China (Fig. 1d), which corresponded well with the locations of the drought (Fig. 1b). Furthermore, this anomalous meridional overturning pattern indicated the northerly currents in the lower troposphere between 30° and 40°N, which also led to the weakening of the EASM and enhancement of the North China drought.

To compare the 2015 extreme drought with those in the history, a precipitation index (PI) was defined in North China (see Methods for details). According to the definition, seven extreme droughts (PI < −1) during 1979–2015 were selected, including 1991, 1999, 2001, 2002, 2006, 2014 and 2015. It is noteworthy that six of them took place in recent decade, but only the 2015 summer drought occurred with a strong El Niño. During another two big El Niño events (i.e., 1982/83, 1997/98), there were wet or moderately dry condition in North China (Fig. 1e). Therefore, a strong El Niño does not necessarily mean an extreme summer drought in North China. However, the circulation backgrounds are similar for the extreme droughts: the positive EU-like circulation anomalies robustly extended from Europe to East Asia along a northwest-southeast orientation, with anomalous high pressure around Lake Baikal and low pressure centered in Ural Mountains and eastern East Asia, despite there were some differences for the meridional structure along the coast region of East Asia (Fig. 1c, Fig. S1).

**Figure 2:**
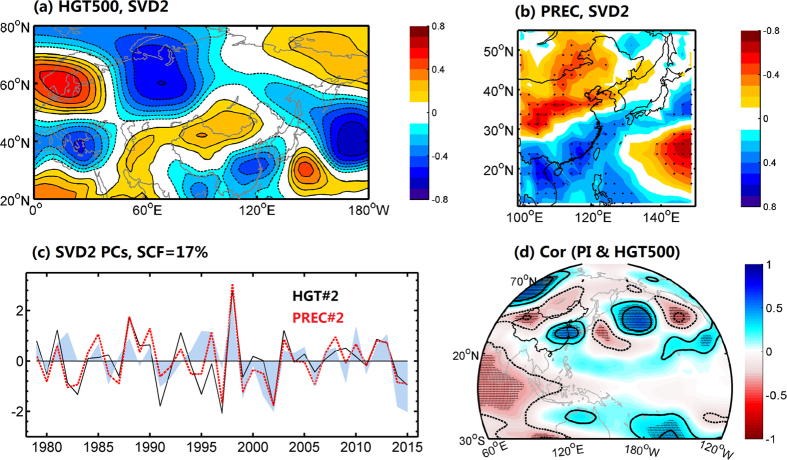
Teleconnection pattern related to the extreme summer drought in North China. (**a** and **b**) are the heterogeneous correlation maps of the second SVD mode for the detrended and normalized 500 hPa geopotential height and PREC precipitation during JA of 1979–2015, respectively. (**c**) The corresponding normalized time series for the second mode of SVD, and the shaded blue curve is the time series of precipitation index (PI) defined in Fig. 1. (**d**) The correlations of the PI and 500 hPa geopotential height for the period of 1979–2015, and the stippling indicates a 95% confidence level according to a two-tailed Student’s t-test. Maps were produced using Matlab version R2012a software (http://www.mathworks.com).

To further identify the robustness of the relationship between the extreme summer drought in North China and the positive EU pattern, we performed the single value decomposition (SVD) analysis on the detrended and standardized 500 hPa geopotential height and precipitation during 1979–2010 (Fig. 2a–c). The heterogeneous correlations for the second mode of SVD showed a northwest-southeast wave-train across Eurasia (Fig. 2a), which closely resembled the EU pattern. Its associated temporal variation also showed highly consistency with the EU index (see Methods), with the correlation coefficient of 0.84 (p < 0.01) (not shown). Correspondingly, there were significant precipitation deficits across North China (Fig. 2b). Meanwhile, the relevant temporal evolutions for the geopotential height and precipitation were highly correlated with a coefficient of −0.86 (p < 0.01). Both SVD temporal series correlated well with the observed precipitation variations over North China (Fig. 2c), with the correlations of −0.53 (p < 0.01) and 0.7 (p < 0.01). It is suggested that the second mode that is closely related with the EU pattern can well represent the variability of summer precipitation across North China. Moreover, the correlation map of PI and geopotential height at 500 hPa for summer (Fig. 2d) also exhibited an obvious zonal wave train as mentioned above.

### Predictability of the 2015 extreme summer drought

**Figure 3:**
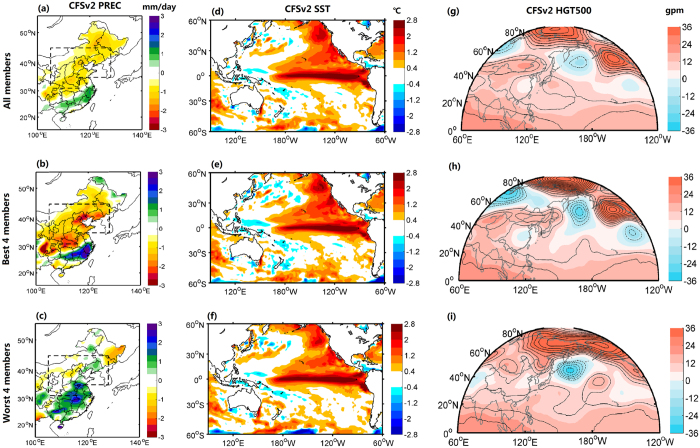
Spatial distributions of CFSv2 predicted anomalies of precipitation, SST and 500 hPa geopotential height in the summer of 2015. The left panels are the composite of (**a**) all ensemble members, (**b**) four best and (**c**) four worst members for 0.5-month lead seasonal forecasts of precipitation anomaly (mm/day). The middle (**d**–**f**) and right panels (**g**–**i**) are the same as the left, but for the anomalies of SST (°C) and 500 hPa geopotential height (gpm). Maps were produced using Matlab version R2012a software (http://www.mathworks.com).

Figure 3 shows the mean July-August (JA) anomalies of precipitation, SST and circulation patterns predicted by NCEP’s Climate Forecast System version 2 (CFSv2)^26^ at 0.5-month lead (see Methods) during the 2015 extreme summer drought period. Compared with the observed precipitation, the CFSv2 model roughly captured the rainfall deficit pattern across North China, but with a weaker intensity (Fig. 3a). In fact, there was a good coherence between the predicted and observed precipitation anomalies in North China during 1982–2015, with a correlation coefficient up to 0.71 (p < 0.01) (not shown).

For the SST prediction during JA of 2015, CFSv2 well captured the El Niño pattern as well as its magnitude (Fig. 3d), which was not surprising because of high predictive skill for NINO3.4 SST by CFSv2^28^,^29^. As for the circulation prediction, although there were some discrepancies as compared with observation, it successfully predicted the negative pressure center over Ural Mountain and the opposite anomaly around Lake Baikal (Fig. 3g). East of the high pressure center of Baikal region, the pressure anomaly was relatively lower than the surrounding, and therefore induced a weak cyclone over eastern part of East Asia, which was consistent with observation. Overall, CFSv2 generally captured the positive EU circulation pattern across Eurasia but with a weaker amplitude, which led to a well-captured drought pattern but with a underestimation of intensity.

To investigate the role of EU pattern in the predictability of extreme summer drought across North China, four best and four worst CFSv2 ensemble members in terms of precipitation predictive skill (see Methods) were selected from the total 24 members (Fig. 3b,c). For the predicted SST anomalies, there were little difference between the best and worst cases, and both agreed with the observed SST anomaly well (Fig. 3e,f). This suggests that a strong El Niño signals can be predicted by the dynamical climate forecast model quite well, but it does not necessarily mean that it will increase the predictability of the extreme summer drought in North China. In fact, the composite of the four best members was more skillful than the full ensemble mean in terms of the prediction of the positive EU pattern (Fig. 3h), while the four worst composite totally failed to capture such circulation pattern (Fig. 3i) and thus missed the drought (Fig. 3c).

### A dynamical-statistical drought forecasting approach

**Figure 4:**
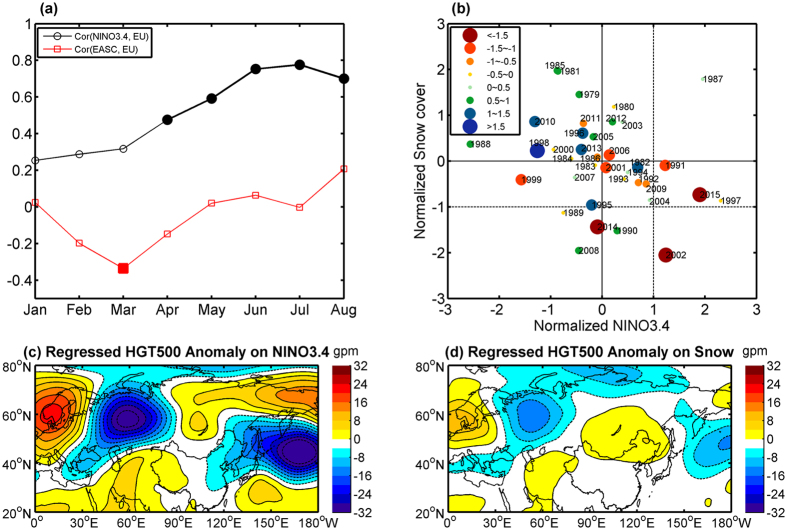
Relationships between ENSO/Eurasian snow cover and the Eurasia teleconnection (EU) pattern. (**a**) Lagged correlations of the Precipitation Index (PI) defined in Fig. 1 and the preceding NINO3.4 index (black) and Eurasian snow cover (red) after detrending. The solid marker indicates the correlation is significant with the confident level greater than 95%. (**b**) Scatter diagram of PI associated with different values of July NINO3.4 index and March Eurasian snow cover. (**c**) Distribution of 500 hPa geopotential height regressed with detrended July NINO3.4 index. (**d**) The same as (**c**), but for the regression with negative March Eurasian snow cover. Maps were produced using Matlab version R2012a software (http://www.mathworks.com).

Given that the dynamical forecast model underpredicted the drought severity, a combination with a statistical method that linked terrestrial/oceanic precursors with the EU pattern might be necessary for a more skillful prediction. For North China, there were significant correlations between NINO3.4 index since preceding April and EU pattern during JA (Fig. 4a and Table S1). The developing of El Niño could arouse a positive EU pattern, characterized with a “-+-’’ wave train from Europe to eastern Asia, or even to northern Pacific (Fig. 4c). Based on July NINO3.4 index that had the highest correlation with the EU pattern index than other preceding months, the regressed PI had a correlation of 0.4 (p < 0.05) with observed PI.

The El Niño is just one of major factors inspiring such drought-prone circulation anomaly across North China. For example, among seven extreme summer droughts hitting North China since 1979, four occurred without strong El Niño signal, one of them even took place in a strong La Niña year (i.e., 1999; Fig. 4b). Besides El Niño, the Eurasian snow cover anomaly in spring^20^,^21^,^22^ as well as Arctic sea ice^30^,^31^, were also considered as important factors inspiring the summer EU-like wave train pattern, through altering the surface albedo and the soil moisture, etc, thereby affecting the EASM and contributing to the drought in North China. The Arctic sea ice had an insignificant correlation with PI, therefore, only the Eurasian snow cover was considered in this study. It was found that Eurasian snow cover in March had the highest correlation with EU pattern in JA, with a detrended correlation coefficient of −0.34 (p < 0.05; Fig. 4a and Table S1). This implies that less Eurasian snow cover in preceding March corresponds to a positive EU pattern with anomalous high pressure around Lake Baikal and its neighbouring region and low pressure in high-latitude Europe, as indicated in Fig. 4d. When combining Eurasian snow cover in March with NINO3.4 index in July, the correlation between the regressed and observed PI was improved significantly from 0.4 (p < 0.05) to 0.5 (p < 0.01; Fig. S2a).

Therefore, observed Eurasian snow cover in March was merged with CFSv2 predicted NINO3.4 index in July to develop a dynamical-statistical forecasting approach by using multivariate linear regression in a cross validation mode (see Methods). Here, the July NINO3.4 index was well predicted by CFSv2 model at 3.5-month lead, with a correlation of 0.67 (p < 0.01; not shown). Figure S2b showed that the approach performed better than CFSv2 dynamical forecast at a lead time of 3.5-month (forecasts starting from the end of March), with an insignificant correlation of 0.19 (p = 0.29) increased to 0.48 (p < 0.01). This suggested a more reliable drought prediction with a dynamical-statistical method at long lead, which would be more useful for drought mitigation.

## Discussion

A drought is always associated with persistent or persistently recurring circulations that produce little or no precipitation. The location and intensity of a drought is directly determined by the large-scale atmospheric circulation anomaly^12^,^13^ rather than SST anomaly, despite the latter is the primary cause and predictability of droughts around the world. In present study, we provide robust evidence that the extreme summer droughts across North China is accompanied with a positive EU pattern, which is favorable for anomalous northerly currents, weakening the EASM, thereby inducing extreme droughts over North China. The dynamical seasonal forecast by using CFSv2 indicates that a skillful forecast of the extreme summer drought across North China is dependent on whether the model captures the positive EU pattern, especially the positive pressure anomaly around Lake Baikal and the negative pressure anomaly around its east or southeast. In addition to the extreme summer droughts across North China, the positive EU pattern represented by quasi-stationary 500 hPa positive height anomalies also results in heat waves through dynamically producing subsidence, clear skies, light winds, warm-air advections, and prolonged hot conditions at the surface^32^,^33^,^34^.

It should be noticed that El Niño can increase the probability of summer droughts in North China, but it does not imply that all extreme droughts in the region require a strong El Niño forcing, such as the summers in 1999 and 2014. Meanwhile, a strong El Niño does not always result in an extreme summer drought, such as summers in 1982 and 1997. Added to El Niño is the high-latitude snow cover, especially under a warming climate. The decreasing spring snow cover in Eurasia has been considered as one of possible reasons for the increasing severe droughts in North and Northeast China in recent decades^22^,^35^. In fact, many weather events during 2015/16 monster El Niño are opposite to those predicted surprisingly, which are mainly due to the influence of the Arctic^36^. Therefore, the change and variability of high-latitude signals should be considered as important factors, and optimally combing them with the low-latitude signals (e.g., El Niño) will enhance the predictability of extreme droughts.

## Methods

### Observation and reanalysis data

Monthly gridded precipitation data at 2.5-degree resolution used in this study was NOAA’s Precipitation Reconstruction (PREC) data (http://www.esrl.noaa.gov/psd/data/gridded/data.prec.html)^37^. Based on PREC data, Precipitation Index (PI) was defined as normalized and area-weighted mean precipitation anomaly averaged over the boxes of (35°N–45°N, 116°E–128°E) and (31°N–45°N, 105°E–116°E) during July-August (JA) to represent the intensity of the summer drought across North China. Seven extreme summer droughts were selected from 1979 to 2015, including 1991, 1999, 2001, 2002, 2006, 2014 and 2015, when the precipitation index (PI) was less than −1. Monthly mean atmospheric data (i.e., geopotential height, u-wind, v-wind, omega) at a horizontal resolution of 1° × 1° was derived from the ERA-Interim^38^ from 1979 to 2015. Sea surface temperature (SST) data used here was NOAA Extended Reconstructed SST version 3 (ERSSTv3) data^39^.

### Definitions of Climate Indices

Several climate indices were used to facilitate the analysis: Niño3.4 index was defined as the average SST anomaly in the region bounded by 5°N to 5°S, from 170°W to 120°W based on ERSSTv3 dataset; and Eurasia snow cover index was obtained from Rutgers University Global Snow Lab^40^ (http://climate.rutgers.edu/snowcover/table_area.php?ui_set=2).

Moreover, the summer EU pattern index was defined as the leading Empirical Orthogonal Functions (EOF) mode of the mean July–August 500 hPa geopotential height (Fig. S3)^41^,^42^. Here the positive EU pattern corresponded to anomalous low pressures over the Ural Mountains region and eastern East Asia, and high pressure between them around Lake Baikal, i.e., a “-+-’’ wave train extending from northern Europe southeastwards to eastern East Asia and its adjacent sea.

### CFSv2 seasonal hindcasts and real-time forecasts

The ensemble hindcast and forecast data from Climate Forecast System version 2 (CFSv2) that was operational at NCEP since March 2011^26^, was also use here. It had 24 ensemble members and was widely used for subseasonal to seasonal forecasting^26^,^28^,^43^,^44^. The four best and four worst members out of the total 24 members were selected based on anomaly correlation^44^ between the observed precipitation anomaly and individual predictions in North China. All the monthly anomalies were based on the climatology from the entire hindcast period (1982–2010). The 0.5-month lead forecasts started from the mid-June, and predicted through July-August (JA). Similarly, the 3.5-month lead forecasts for the JA started from the mid of March.

### Dynamical-statistical forecasting approach

A dynamical-statistical forecasting approach based on CFSv2-predicted NINO3.4 index and observed Eurasian spring snow cover was developed to predict PI by a linear regression in a cross validation mode. For each summer *s*, we established a bivariate linear regression equation of PI based on July NINO3.4 predicted by CFSv2 at 3.5-month lead time and observed Eurasian snow cover in preceding March in the remaining years: PI_(−*s*)_ = a_*s*_ × NINO3.4_(−*s*)_ + b_*s*_ × Snow_(−*s*)_ + c_*s*_, where *s* indicated a given summer, the variables with the subscripts (−*s*) indicated information excluding the target summer *s*, a_*s*_, b_*s*_ and c_*s*_ were the corresponding regression coefficients. The dynamical-statistical hindcasts of PI in summer *s* was calculated in such a cross validation mode.

## Additional Information

**How to cite this article**: Wang, S. *et al*. Does a Strong El Niño Imply a Higher Predictability of Extreme Drought? *Sci. Rep.* **7**, 40741; doi: 10.1038/srep40741 (2017).

**Publisher's note:** Springer Nature remains neutral with regard to jurisdictional claims in published maps and institutional affiliations.

## Supplementary Material

Supplementary Information
