# Supplementary material for: Does a Strong El Niño Imply a Higher Predictability of Extreme Drought?
Source: Sci Rep. 2017 Jan 17;7:40741. doi: 10.1038/srep40741 (PMC5240109; doi:10.1038/srep40741)
Supplement: Supplementary Information [file srep40741-s1.doc]

*Supplementary Information to*

**Does a Strong El Niño Imply a Higher Predictability of Extreme Drought?**

Shanshan Wang^1,2^, Xing Yuan1[[1]](#footnote-2)*, Yaohui Li^2^

^1^RCE-TEA, Institute of Atmospheric Physics, Chinese Academy of Sciences, Beijing 100029, China

^2^Key Laboratory of Arid Climatic Change and Reducing Disaster of Gansu Province, and Key Open Laboratory of Arid Climate Change and Disaster Reduction of CMA, Institute of Arid Meteorology, CMA, Lanzhou 730020, China

Submitted to *Scientific Reports*

Revised 30 November 2016


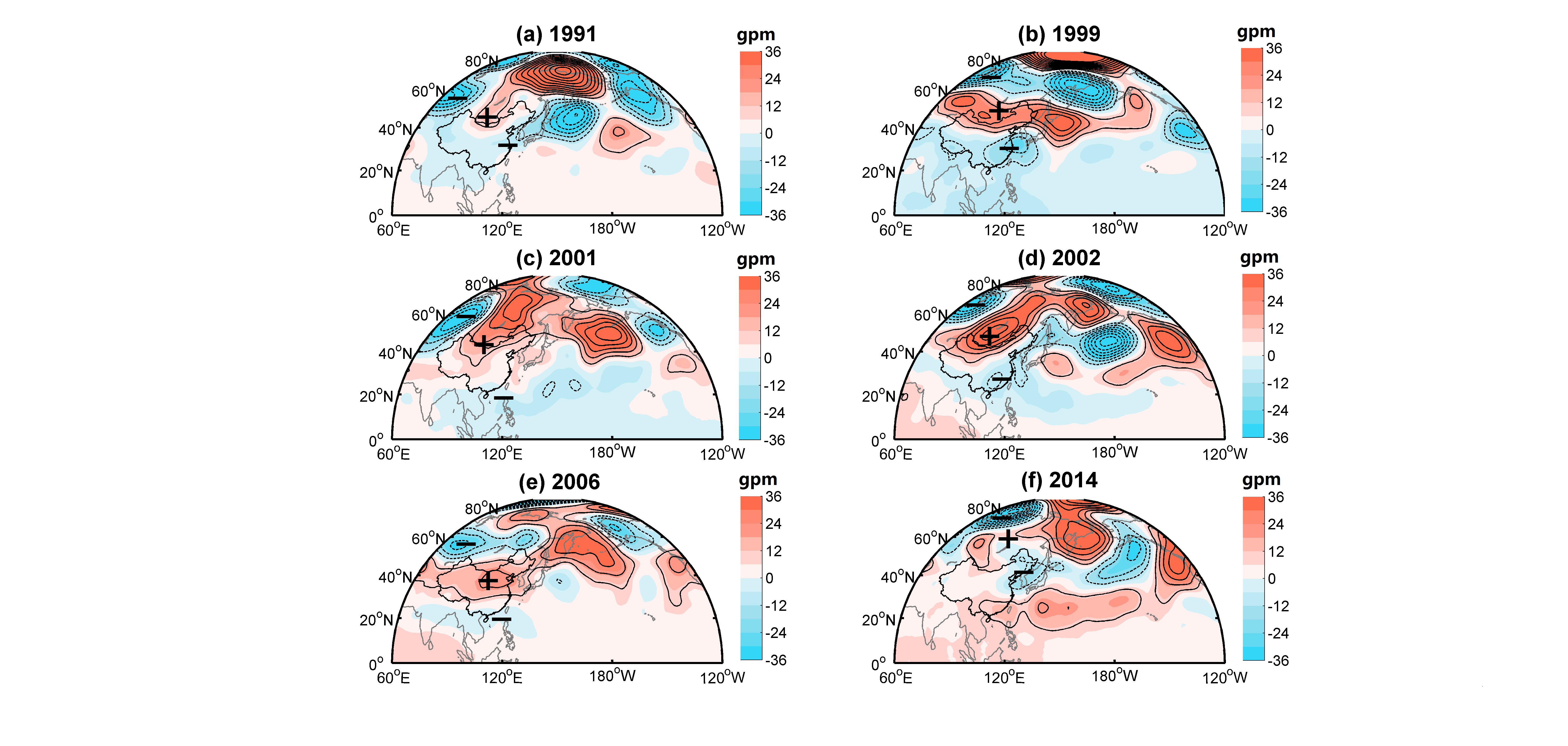


**Figure S1** **| Anomalies of 500 hPa geopotential heights during North China extreme summer drought periods.** Six historical extreme summer droughts are selected for 1991, 1999, 2001, 2002, 2006 and 2014 when the precipitation index (PI, see Methods for definition) is less than -1. Maps were produced using Matlab version R2012a software (http://www.mathworks.com).


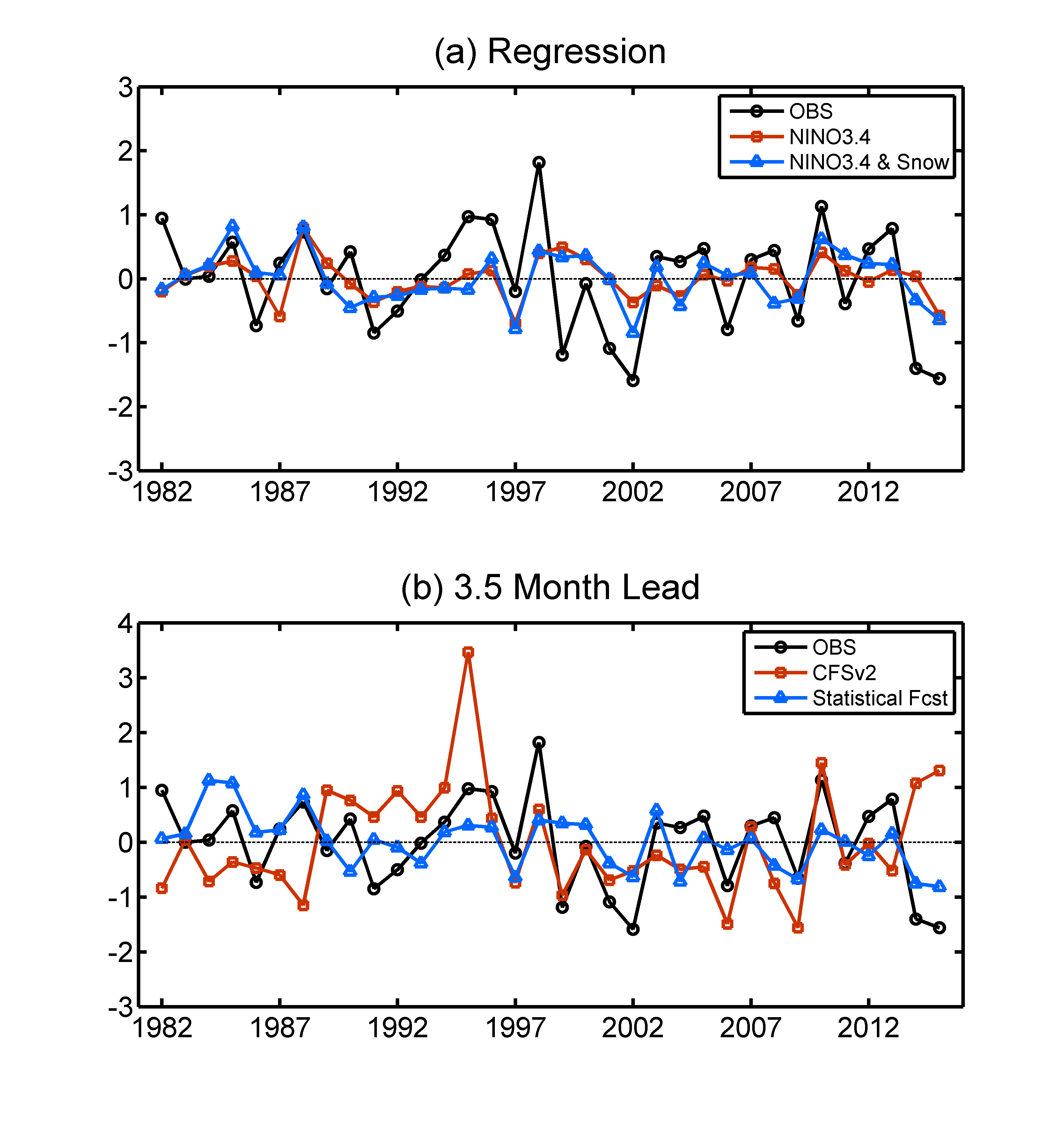


**Figure S2 |** **Time series of observed, regressed and predicted precipitation index (PI).** (a) NINO3.4 denotes the regressed PI (red) against the observed NINO3.4 alone, and NINO3.4 & Snow (blue) is against these two factors. (b) CFSv2 (red) represents dynamical climate forecasts averaged from 24 ensemble members. Statistical forecasts (blue) are made by linearly regressing PI against the NINO3.4 predicted by CFSv2 at 3.5-month lead and the observed Eurasian snow cover index in March, and a cross validation procedure is used to estimate the regression equations (see Methods for details).


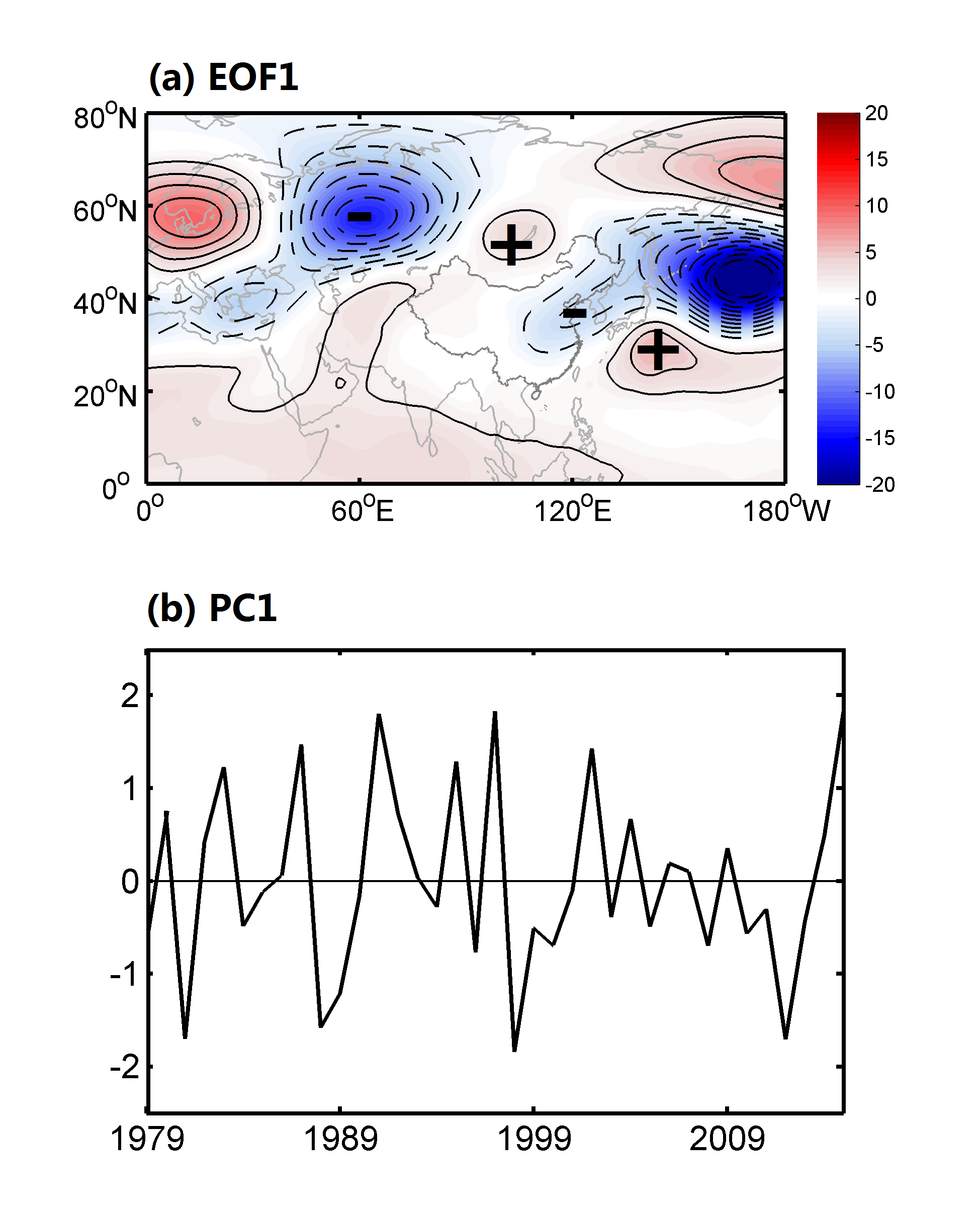


**Figure S3 | Spatial and temporal variations of the leading EOF mode of summer 500 hPa geopotential height for the period of 1979-2015.** The leading mode explains 18.3% of the variance with negative anomalies over the Ural mountain and East Asia coast, and a positive anomaly around Lake Baikal, which represents the positive Eurasia teleconnection (EU) pattern. The corresponding time series is used here to characterize the amplitude of the summer EU pattern. Maps were produced using Matlab version R2012a software (http://www.mathworks.com).

**Table S1 | Correlation coefficients between indices.** Eurasia teleconnection (EU) pattern index and Precipitation Index (PI) are defined by using the means for July–August, NINO34 is for the July, and Eurasia snow cover index (Snow) is for the preceding March (see Methods for details). * and ** indicate the 95% and 99% confidence levels based on the Student’s t test. All correlation coefficients based on the detrended indices.

|  | **EU** | **NINO34** | **Snow** |
| --- | --- | --- | --- |
| **PI** | -0.52** | -0.39* | 0.34* |
| **EU** |  | 0.74** | -0.34* |

1. ^*^*Corresponding author address:* Xing Yuan, RCE-TEA, Institute of Atmospheric Physics, Chinese Academy of Sciences, Beijing 100029, China. E-mail: yuanxing@tea.ac.cn [↑](#footnote-ref-2)
